# Significance of electronic health records: A comparative study of vaccination rates in patients with sickle cell disease

**DOI:** 10.12669/pjms.333.12837

**Published:** 2017

**Authors:** Asli Korur, Süheyl Asma, Cigdem Gereklioglu, Soner Solmaz, Can Boga, Akatlı Kürsat Ozsahin, Altug Kut

**Affiliations:** 1 Asli Korur M.D. Department of Family Medicine, Adult Bone Marrow Transplantation Unit, 01250, Adana, Turkey. Baskent University Faculty of Medicine, Yuregir, 01250, Adana, Turkey; 2 Suheyl Asma M.D. Assistant Professor, Department of Family Medicine, Adult Bone Marrow Transplantation Unit, 01250, Adana, Turkey. Baskent University Faculty of Medicine, Yuregir, 01250, Adana, Turkey; 3 Cigdem Gereklioglu M.D. Department of Family Medicine, Adult Bone Marrow Transplantation Unit, 01250, Adana, Turkey. Baskent University Faculty of Medicine, Yuregir, 01250, Adana, Turkey; 4 Soner Solmaz M.D. Associate Professor, Department of Hematology, Adult Bone Marrow Transplantation Unit, 01250, Adana, Turkey. Baskent University Faculty of Medicine, Yuregir, 01250, Adana, Turkey; 5 Can Boğa M.D. Professor, Department of Hematology, Adult Bone Marrow Transplantation Unit, 01250, Adana, Turkey. Baskent University Faculty of Medicine, Yuregir, 01250, Adana, Turkey; 6 Akatlı Kursat Ozsahin M.D. Associate Professor, Baskent University Faculty of Medicine, Yuregir, 01250, Adana, Turkey; 7 Altug Kut M.D. Professor, Baskent University Faculty of Medicine, Mutlukent Mah 1963 sok No: 17 Ümitköy, Ankara, Turkey

**Keywords:** Electronic health records, Sickle cell disease, Vaccination

## Abstract

**Objective:**

In this study, we investigated the influence of electronic health records (EHR) and electronic vaccination schedule applications on the vaccination status of patients who were admitted to our Center for the treatment of sickle cell disease (SCD).

**Methods:**

The vaccination status against influenza and pneumococcus infection was determined in 93 patients who were admitted to the hematology outpatient clinic, Baskent University Adana Hospital from April 2004 to March 2009. The vaccination status was then re-evaluated following establishment of EHR and electronic vaccination schedules in 2012.

**Results:**

Of the 93 patients with SCD 21.5% (n = 20) were vaccinated against pneumococcus and 21.5% (n = 20) were regularly vaccinated against influenza. When the vaccination rates of 59 of 93 patients who presented for their regular control examinations were analyzed following establishment of EHR and vaccination schedules in 2012, these rates were 49.2% (n = 29) and 50.8% (n = 30) for influenza and pneumococcus, respectively, after EHR; there were 23.7% (n = 14) and 20.3% (n = 12), respectively, before EHR. A statistically significant difference was found between the vaccination rates before and after EHR (p < 0.05).

**Conclusion:**

Although viral and bacterial infections are life-threatening health problems in patients with SCD, the vaccination rates were low in high-risk patients. However, these rates increased after application of electronic vaccination schedules.

## INTRODUCTION

Sickle cell disease (SCD) is the most common hemoglobinopathy worldwide. It is prevalent in Africa, India, and Mediterranean countries, such as Greece, Turkey, and Italy. The disease is symptomatic only in homozygous individuals.^1^ SCD causes chronic hemolysis, hemolytic crisis, and infections that may precipitate hemolysis.^2^

Progressive vaso-occlusive damage to the spleen leads to a state of functional asplenia together with other immune system dysfunctions.^3^ People with SCD are at high risk for complications of influenza and pneumococcal infections because they are susceptible to bacterial infections caused by encapsulated organisms (including *Streptococcus pneumoniae* and *Haemophilus influenzae*) due to their impaired splenic function, increased airway hyperactivity, and risk of acute chest syndrome following respiratory infections.^4^ Vaccination against pneumococcus, influenza, and haemophilus influenzae Type-B (HiB) is recommended for patients with SCD.^5^-^7^

Electronic health records (EHR) enable us to maintain records that cannot be created on paper. These include reminders for both patients and physicians about preventive health measures as well as ways to monitor the achievement of preventive healthcare goals, such as vaccination rates.^8^ Studies have shown improvements in preventive health services with the use of electronic reminder systems.^9^ In this study, we investigated the influence of EHR and electronic vaccination monitoring on vaccination rates against influenza and pneumococcus in patients with SCD.

## METHODS

Data from 93 patients who were admitted to the Hematology Department of Baskent University Adana Hospital from April 2004 to March 2009 with a diagnosis of SCD and aged 16 to 58 years (64.6% female, 35.4% male) were included in this study. The sociodemographic data for the patients were obtained from the patients’ files.

### Sample Selection

The rates of vaccination against influenza and pneumococcus before 2012, when EHR were not being used, were compared with those after 2012. For this purpose, we re-analyzed the 2012 vaccination rates against influenza and pneumococcus for 93 patients (60 female, 33 male). However, 8 patients died and 26 did not present for their regular control examinations; these patients were excluded from the analysis. Thus, we evaluated the remaining 59 patients, and a second analysis was performed to compare the vaccination rates of these 59 patients before and after 2012. The only exclusion criterion was an age of <18 years.

### Data Collection

Electronic vaccination monitoring cards containing data on the date of vaccination were used to follow up with outpatients with SCD beginning in 2012. This record system was designed to notify the physicians.

### Records and Data Security

The patients’ data were obtained from the Data Management System of the hospital (Nucleus version 9.3.39; Monad Software Company, Ankara, Turkey). Anemnesis forms and vaccination follow-up cards were created specifically for patients with SCD in this system to ensure inclusion of the vaccinations recommended by the World Health Organization (mainly influenza and pneumococcus). The vaccination status was evaluated when the patient was admitted to the outpatient clinic for any reason. Prior vaccinations were also recorded electronically. All patients who had not been previously vaccinated were given a copy of the vaccination card, which showed the scheduled dates of vaccinations. This software works in accordance with the relevant standard operating procedures for data authorization, backup, storage, and security, and the accuracy of the data is checked by the Data Audit Council assigned by the hospital management.^10^

### Statistical Analyses

Data were analyzed using the SPSS 17.0 statistical package program (SPSS Inc., Chicago, IL, USA). Parametric data were compared using Student’s t test, and non-parametric data were compared using Pearson’s chi-square test and Fisher’s exact test. A p value of <0.05 was considered statistically significant.

### Ethical Committee

Ethics Committee approval was obtained from Baskent University (KA17/45).

## RESULTS

The initial audit and re-audit identified 93 patients (33 male, 60 female) with a mean age of 31.3 ± 9.7 (range, 16-58) years and 59 patients (21 male, 38 female) with a mean age of 29.0 ± 8.4 (range, 19-54) years. Among the 93 patients who were admitted to the outpatient clinic from 2004 to 2009, 21.5% (n = 20) were vaccinated against pneumococcus and 21.5% (n = 20) were regularly vaccinated against influenza.

According to the records of 59 patients who presented for their regular control examinations, 12 (20.3%) had been vaccinated for pneumococcus and 14 (23.7%) had been vaccinated for influenza before 2012; these rates were 49.2% for influenza and 50.8% for pneumococcus after 2012. A statistically significant difference was detected between these two time periods (p < 0.05).

## DISCUSSION

Because pneumococcal and influenza vaccines are the most frequently administered and updated, this study primarily focused on these two vaccines. The vaccines administered in accordance with the childhood vaccination schedule (HiB, hepatitis B virus, andmeningococcus Group-C [Men-C] are often not recorded in adult vaccination records.

**Table-I:** Comparison of vaccination rates before and after establishment of EHR.

*Vaccine*	*Vaccination Rate*				

	*Before EHR*		*After EHR*		*P*

	*n*	*%*	*n*	*%*	
Influenza	14	23.7	29	49.2	0.005
Pneumococcus	12	20.3	30	50.8	0.001

Given that patients with SCD are more susceptible to infection, particularly by *S. pneumonia* and HiB, due to splenic dysfunction, the inability to identify patients at high risk for these potentially fatal infections is a barrier to improvements in vaccination rates. Influenza, pneumococcus, and HiB vaccines have also been shown to be safe and effective in patients with SCD.^7^,^11^-^13^ Pneumococcal immunization should begin with locally available pneumococcal conjugate vaccines (7, 10, or 13 valency) and supplemented at ages 2 and 5-7 years with the 23-valent pneumococcal polysaccharide vaccine. HiB series, meningococcal conjugate vaccine, hepatitis B, and yearly influenza vaccinations are also recommended.^5^-^7^

Infections in patients with SCD may lead to painful crises, hospitalization, and mortality. According to a study performed in the Eastern Mediterranean region by Karacaoglu et al., the most common causes of death were acute chest syndrome, followed by splenic sequestration and prolonged painful crisis-related multi-organ failure.^14^ The researchers found that the mean age at death was 34.1 ± 10 (range, 18-54) years for males, 40.1 ± 15 (range, 17-64) years for females, and 36.6 ± 13 (range, 17-64) years overall. Bundy et al.^6^ found that children with SCD were hospitalized due to influenza at a 56-fold higher rate than children without SCD. Pneumococcal bacteremia is particularly severe in young children. The risk persists in adults, especially for nosocomial infections.^15^ Seventy percent of cases of septicemia and meningitis among patients aged <5 years with SCD are caused by *S. pneumonia*.^16^

Patients with SCD in the UK and USA are placed on prophylaxis and vaccinated against *H. influenza* and *Pneumococcus* sp.^17^,^18^ Twenty-five children aged ≤16 years were analyzed in a study of 58 patients with SCD in the UK, and 14 (56%) were found to have completed immunization against pneumococcus, HiB, and MenC, with 8 (32%) having some coverage and 3 (12%) having none at all. Among 33 adults, only 4 patients (12%) had completed coverage, with up-to-date coverage against pneumococcus in 21%, HiB in 18%, and MenC in 15%. Current immunization against viral influenza was found in only 12% of adults and 8% of children, which are rates similar to those found in other UK studies.^18^ The National Confidential Enquiry into Patient Outcome and Death (NCEPOD) study revealed 6% influenza, 32% pneumococcal, 19% MenC, and 23% HiB vaccine uptake levels.^19^,^20^ Similarly, an investigation of pneumococcal vaccine uptake in 3584 patients with either SCD or celiac disease revealed only 15.9% uptake in the five years preceding 2005.^21^ This study demonstrated a low level of vaccination uptake in patients with SCD in this region. Only 23.7% of patients had an up-to-date influenza vaccine, and 20.3% had a current pneumococcal vaccine in the initial study.

Many techniques have been used to improve the vaccination rates in patients with SCD throughout the world. A meta-analysis of improvement initiatives for community-dwelling adults showed success with the use of nonphysician personnel, activating patients through direct outreach, and audit and feedback aimed at physicians. Also successful were the use of a non-clinician patient navigator for direct outreach and providing updates to the clinical team on overall vaccination rates throughout the project.^22^ Although that study revealed the importance of measures aimed at improving vaccination rates, we could not make a comparison because EHR were not used in that study.

The Healthy People 2020 vaccination goals can be improved by EHR.^23^ The use of a database to target reminders to high-risk patients, followed by a recall letter for those remaining unvaccinated, was shown to increase influenza vaccination rates in a group of pediatric practices.^24^ Through the use of an alert, EHR resulted in an increase in influenza vaccination rates from 47% to 65% in a study of immunocompromised patients with rheumatologic diseases.^25^ Another study of 2-year-old patients with SCD revealed that an EHR-based clinical alert intervention was associated with increases in captured opportunities for vaccination at both sick and well visits and detected significant improvements in immunization rates. As EHRs become more common in medical practice, such systems may transform immunization delivery to children.^26^ A study conducted by Asma^27^ with similar patient groups in the same region as our study revealed a pneumococcus immunization rate of 21.5% and an influenza immunization rate of 22.5% in adult patients with SCD before commencement of EHR for vaccination. We found these rates to be 50.8% and 49.2% for pneumococcus and influenza, respectively. Our study indicates that the Healthy People 2020 goal for influenza vaccination rates could be achieved for a medically and socially high-risk population through the use of enhanced EHR. Vaccination of adults with chronic diseases or hemoglobinopathy is important and in accordance with the core competencies of family medicine, such as improvement of health and wellness through appropriate and effective interventions.^28^

### Limitations of the study

First, the sample size was relatively small. Second, the study was conducted in one region, and the results cannot be assumed to represent all patients with SCD in Turkey. The potential causes of low vaccination rates may include insufficient knowledge among primary care physicians about this patient group; physicians may not volunteer for follow up of these patients and may refer them to tertiary care facilities where hematologists work. On the other hand, the high costs of tertiary care for chronic diseases may lead to low vaccination rates due to reduced tertiary care admissions. Consequently, these patients may be lost to follow up. Problems with transportation to specialized centers may be another factor that leads to low admissions and, thus, the inability to achieve sufficient vaccination rates. The media also influence people’s decisions, beliefs, and attitudes about vaccination.

## CONCLUSIONS

Viral and bacterial infections are an important cause of mortality and morbidity in patients with SCD. Studies of this patient group have shown insufficient vaccination rates. We believe that vaccination rates may improve through the use of vaccination cards, EHR, and regular follow up during vaccination periods.

### Authors Contribution

**AK, SA, SS:** conceived, designed, data collection, editing of manuscript.

**CG, AKO, AK:** data collection and manuscript writing.

**SS, CB, AKO, AK:** did statistical analysis, review and final approval of manuscript.
